# Association between Tooth Loss and Gastric Cancer: A Meta-Analysis of Observational Studies

**DOI:** 10.1371/journal.pone.0149653

**Published:** 2016-03-02

**Authors:** Xin-Hai Yin, Ya-Dong Wang, Hong Luo, Ke Zhao, Guang-Lei Huang, Si-Yang Luo, Ju-Xiang Peng, Ju-Kun Song

**Affiliations:** 1 Department of Oral and Maxillary Surgery, Gui Zhou Provincial People's hospital, Guiyang, China; 2 Department of Orthodontics, Stomatology hospital of Gui yang, Guiyang, China; University of North Carolina at Chapel Hill, UNITED STATES

## Abstract

Observational studies showed that tooth loss is associated with gastric cancer, but the findings are inconsistent. In this study, a meta-analysis was conducted to evaluate the relationship between tooth loss and gastric cancer. Relevant studies were screened in PubMed and Embase databases, and nine observational studies were considered eligible for the analysis. The combined relative risks for the highest versus the lowest categories of tooth loss were 1.86 (95% CI: 1.08–3.21) and 1.31 (95% CI: 1.12–1.53) in case control and cohort studies, respectively. However, unstable results were observed in the stratified and sensitivity analysis. The current evidence, based solely on four case-control studies and five cohort studies, suggested that tooth loss is a potential marker of gastric cancer. However, we can not concluded at this time that tooth loss may be a risk factor for gastric cancer due to significant heterogeneity among studies and mixed results between case-control studies and cohort studies. Additional large-scale and high-quality prospective studies are required to evaluate the association between tooth loss and risk of gastric cancer.

## Introduction

Despite decreased incidence and mortality rates of gastric cancer over the last several decades, this disease remains the fifth most frequently occurring cancer and the third leading cause of cancer-related deaths [1]. According to the latest statistical study in the USA, 24,590 new cases of gastric cancer were diagnosed, and 10,720 deaths caused by this disease were recorded in 2015[1]. Risk factors for gastric cancer include smoking tobacco, heavy alcohol drinking, diets low in fruits and vegetables, diets with excessive salt and nitrates, male gender, and infection with Helicobacter pylori [2]. The most significant etiologic factors include H. pylori infection, smoking tobacco, and heavy alcohol drinking[3]. Therefore, understanding the risk factors and the underlying mechanism of gastric cancer is vital for developing preventive strategies for gastric cancer treatment.

Tooth loss significantly influences mastication, diets, nutrition intake, aesthetics, and food choice. Epidemiological studies have been conducted to determine the association between tooth loss and susceptibility to gastric cancer[4–12]. However, the results of these studies are conflicting and inconsistent. The majority of studies reported that tooth loss is correlated with gastric cancer risk [5, 6, 8–10, 12], whereas other studies failed to demonstrate a significant association[4, 7, 11]. Given the poor prognosis of gastric cancer and common incidence of tooth loss, we conducted a meta-analysis to summarize the association between tooth loss and gastric cancer. Elucidating this relationship may emphasize the importance of preventive methods for gastric cancer.

## Methods

### Literature search

A literature search in PubMed and EMBASE databases was performed until April 2015. The following search terms were used without any limitations: stomach or gastric; cancer, carcinoma, tumor, or tumour; and tooth, teeth, tooth loss, teeth loss, or dental status. References from eligible articles were retrieved.

### Eligibility criteria

Studies were included in the meta-analysis if they met the following criteria: (1) the exposure of interest was tooth loss; (2) the outcome was gastric cancer incidence; and (3) relative risk (RR), odds ratio (OR), or hazard risk (HR) with the corresponding 95% confidence interval (CI) was reported, or raw data for estimating crude OR, RR, or HR with the corresponding 95% CI were available. Moreover, studies on periodontal diseases and gastric cancer risk were excluded if they did not provide information on the number of teeth. If duplicated data were presented in several studies, only the study with the largest sample size was included.

### Data extraction

Two authors (JKS and XHY) independently extracted data from the selected studies by using a standardized extraction form. The following key points were collected: first author’s surname, year of publication, study design, country, duration of follow-up, gender, total number of cases and subjects for cohort studies, total number of cases and controls for case control studies, assessment methods for tooth loss, and multiple adjusted RR, OR, and HR of tooth loss and the corresponding 95% CI for each category of exposure. The adjusted RR was extracted in preference to non-adjusted RR; however, unadjusted RR and CI were calculated when RR was not provided. Disagreements between reviewers regarding data extraction were resolved through discussion.

### Quality assessment

The methodological quality of included studies was evaluated using the Newcastle–Ottawa scale (NOS)[13]. The check list contains nine items for case-control studies and cohort studies, with every item accounting for 1 point. High-quality studies were rated with scores > 6.

### Statistical analysis

This meta-analysis summarized the RR for the highest versus the lowest categories for each study because the categories of tooth loss varied across studies. OR in case-control studies was considered approximations of RR, considering the low absolute risk of gastric cancer. Overall RR was summarized to assess the strength of association between tooth loss and gastric cancer. A random-effects model of DerSimonian and Laird method was used to calculate the summary risk estimates irrespective of heterogeneity, which incorporates both within-study and between-study variabilities[14]. Subgroup analysis was stratified by adjustment for covariates and geographic region. Sensitivity analysis was also conducted by omitting one study in each turn and recalculating the summarized RR for the remaining studies.

Publication bias was evaluated using Begg’s and Egger’s tests (rank correlation and linear regression methods, respectively)[15, 16]. All statistical analysis was conducted using Stata version 13.1 (StataCorp, College Station, TX, USA).

## Results

### Literature search and study characteristics

**Fig 1:**
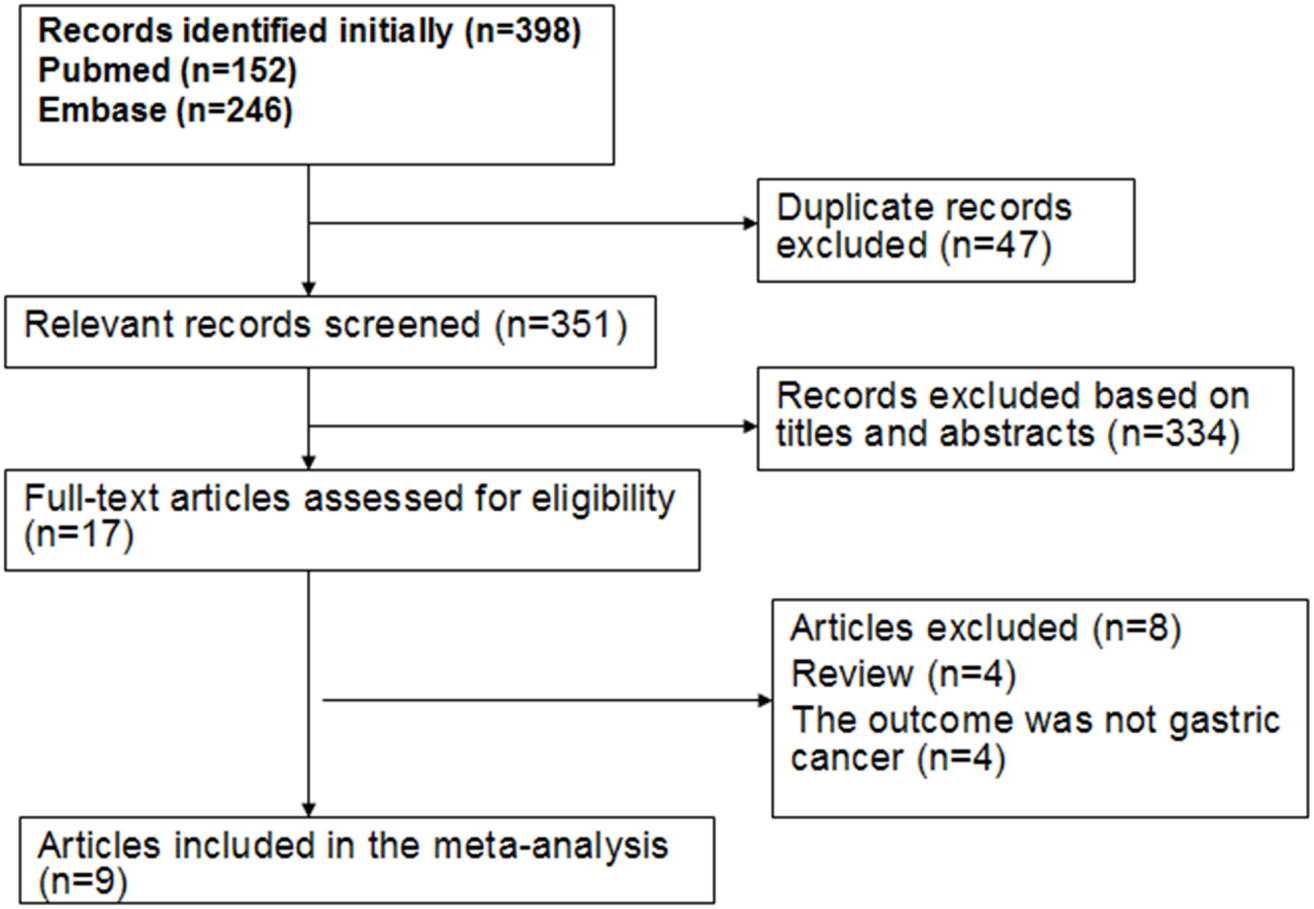
Flow chart of procedures from identification of eligible studies to final inclusion.

A diagram showing the details of study inclusion is shown in Fig 1. A total of 398 studies were screened using the outlined search strategy and selection based on the inclusion criteria; of these studies, 47 were excluded because they were duplicates and 334 were excluded based on their titles and abstracts. Seventeen full-text articles were reviewed for further assessment. Four articles were then excluded because they are narrative reviews[10, 17–19], and another four articles were excluded because their reported outcomes are not related to gastric cancer[20–23]. Abnet et al.[9] conducted two studies on the same Chinese population but reported different incidence end-points. In Ref.[5], the results for risk of gastric cancer were reported, and the follow-up period was from 1985 to 2001[5]; in Ref.[9], the results for risk of gastric cancer subsites were reported, and the follow-up duration was from 1985 to 1991. Therefore, the results for the overall estimate and gastric cancer subsites were included in the analysis. Finally, nine articles (four case controls and five cohort studies) were considered eligible for inclusion in the meta-analysis. Eight articles, which included four case-control studies[6, 7, 10, 12] and four cohort studies[4, 5, 8, 11], reported the association between gastric cancer risk and tooth loss. Moreover, three articles, which included one case-control study[10] and two cohort studies[8, 9], reported the association between risk of gastric cancer subsites and tooth loss.

Tables 1 and 2 show the characteristics of the included case control and cohort studies, which were published from 1990 to 2013. The number of patients with gastric cancer ranged from 10[4] to 866[5] in the cohort studies and from 100[6] to 702[7] in the case-control studies. Five studies were conducted in Asia[5, 7, 9, 10, 12], and two studies were performed in the United States[4, 11] and two in Europe[6, 8]. Most studies reported that cancers were histologically, pathologically, or clinically confirmed or were identified from the national/regional cancer registries, which we assumed verified the cancers histologically. However, tooth loss was assessed using different strategies. Four studies used a questionnaire to classify tooth loss[6–8, 12]. Three studies employed questionnaires and clinical examination as diagnostic criteria[5, 9, 11], and two studies used clinical examination only as the diagnostic criterion[4, 10]. Three studies that examined the association between tooth loss and gastric cancer risk differed in terms of the anatomy of histological subsites[8–10]. Furthermore, two studies were exclusive to men[11], and the remaining studies included both men and women[4–7, 9, 10, 12]. Three studies did not adjust for confounding factors[4, 6, 12], and the five other studies adjusted for traditional risk factors, such as age, sex, and education, as well as other confounding factors in gastric cancer[5, 7–11]. In addition, three studies controlled the adjusted values, such as smoking and alcohol drinking status[5, 7, 10]. Only one study considered the factor of *H*. *pylori* infection[8].

**Table 1:** Characteristic of case-control studies included in the meta-analysis.

Study	Year	Country	Study design	No. of cases	No. of controls	Sex	Outcome ascertainment	Assessment of tooth loss	Follow-up(yrs)	Adjustment for covariates
Demirer T	1990	Turkey	A case-control study	100	200	W and M	Cancers were histologically proven.	Questionnaire	1	Unadjust
Watabe K	1998	Japan	A case-control study	242	484	W and M	Cancers were pathologically confirmed.	Questionnaire	1	Unadjust
Hiraki A	2008	Japan	A Hospital-based case-control study	702	1,404	W and M	Cancers were confirmed by the national cancer registery.	Self-administered questionnaire	4	Adjusted for age, sex, smoking and drinking status (never, former, current), vegetable and fruit intake, BMI, and regular exercise.
Shakeri A	2013	Iran	A case-control study	309	613	W and M	Cancers were histologically proven.	Physical examination by dentists	7	Adjusted for age, ethnicity, education fruit and vegetable use, socioeconomic status, ever opium or tobacco use, and denture use.

NA, not available; M, male; W, female.

**Table 2:** Characteristic of cohort studies included in the meta-analysis.

Study	Year	Country	Study design	No. of patients	Cohort size	Sex	Outcome ascertainmen	Assessment of tooth loss	Follow-up(yrs)	Adjustment for covariates
Abnet CC	2001	China	A prospective cohort study	533	28,868	W and M	Cancers were confirmed by Histology、cytology or X-ray through a panel of expert.	Questionnaire and clinical examination by interviewer	5.25	Adjusted for age sex, tobacco use, and alcohol use.
Hujoel PP	2003	United states	A population based cohort study	10	11,328	W and M	Cancers were confirmed by a medical examination and a standardized medical history, ICD-9.	Periodontal examination by dentists	21	Unadjust
Abnet CC	2005	China	A population-based cohort study	866	29,584	W and M	Cancers were confirmed by medical record and 90% cancers were confirmed by the X-rays, crtology and histology.	Questionnaire and physical examination by interviewer	15	Adjusted for age, sex, ever-smoking, height, weight, and systolic blood pressure
Abnet CC	2005	Finland	A prospective cohort study	245	29,124	M	Cancers (~100%) were identified by the Finish Cancer Register.	Questionnaire	5	Adjusted for age at randomization and education.
Michaud DS	2008	United states	A prospective cohort study	106	48,375	M	Cancers (90%) were confirmed by medical records and pathology reports.	Self-reported and clinical examination	18	Adjusted for age (continuous), race (White, Asian, Black), physical activity (quintiles), history of diabetes (yes/no), alcohol (quartiles), body mass index (<22, 22–24,9, 25–29.9, 30+), geographic location (South, West, Northeast, Midwest), height (quintiles), calcium intake (quintiles), total caloric intake (quintiles), red meat intake (quintiles), fruit and vegetable intake (quintiles), and vitamin D score (deciles) smoking history (never, past quit ≤10 years, past quit >10 years, current 1–14 cigarettes per day, 15–24 cigarettes per day, 25+ cigarettes per day), and pack-years (continuous).

NA, not available; M, male; W, female.

NOS was used to evaluate the quality of the eligible studies (Table 3), and the median NOS score was 6.1 (range of 4–8).

**Table 3:** Quality assessment of included studies based on Newcastle-Ottawa scale.

Author	year	Selection	Comparability	Exposure
Demirer T	1990	2	0	2
Watabe K	1998	3	0	2
Abnet CC	2001	2	1	3
Hujoel PP	2003	3	0	3
Abnet CC (China)	2005	2	1	3
Abnet CC (Finland)	2005	3	2	3
Hiraki A	2008	3	1	2
Michaud DS	2008	3	1	3
Shakeri A	2013	3	1	3

### Tooth loss and overall gastric cancer

**Fig 2:**
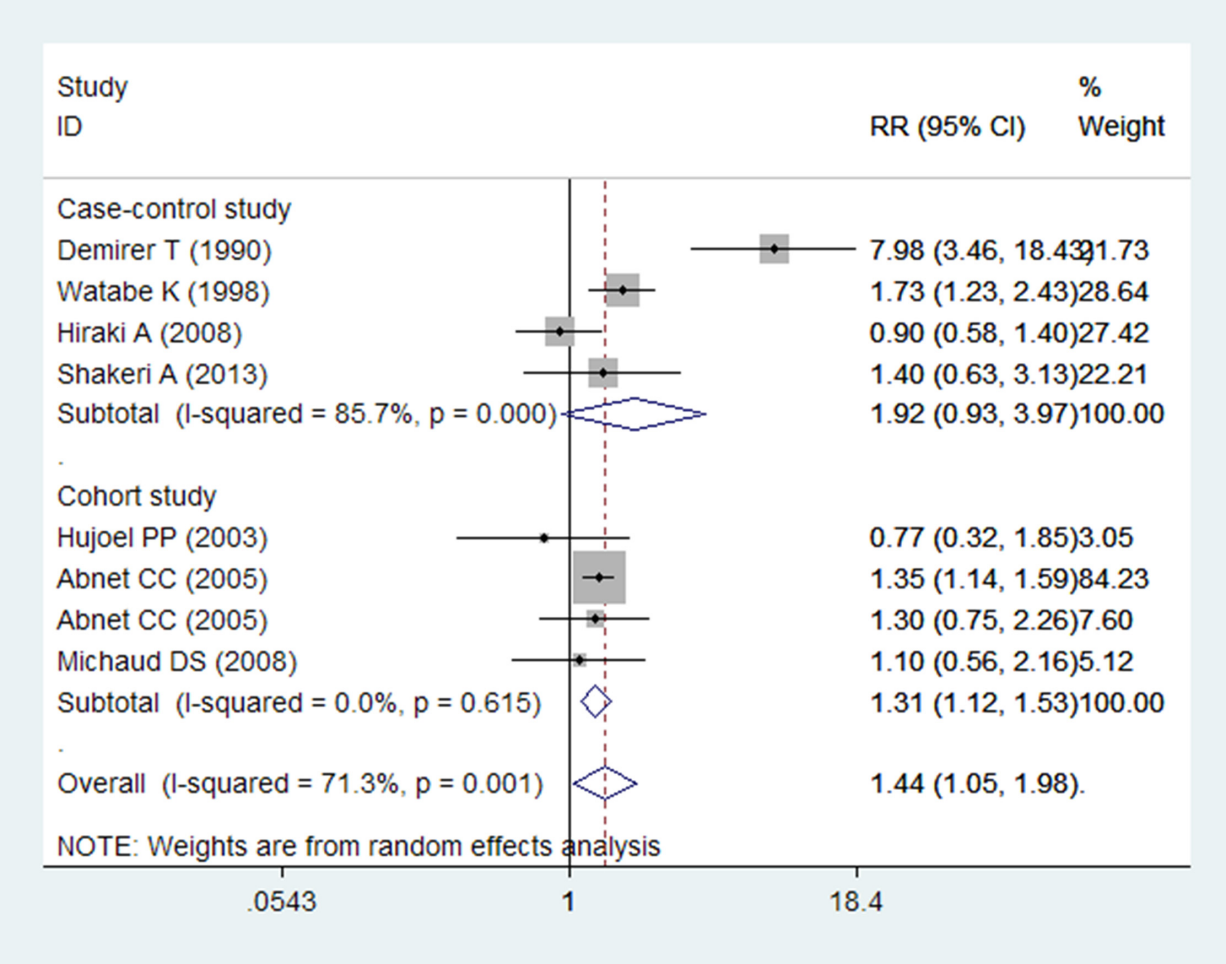
Forest plot for tooth loss and gastric cancer. Studies are pooled with the random-effects model.

In the overall meta-analysis, the summarized RR estimates for the highest versus the lowest categories of tooth loss were pooled to provide a total risk estimate by using the random-effects model (RR = 1.44, 95% CI = 1.05–1.98; P = 0.001). The test for heterogeneity was significant (P = 0.001, *I*^2^ = 71.3%; Fig 2).

The meta-analysis of all the four case-control studies indicated that tooth loss was not associated with increased incidence of gastric cancer (RR = 1.92; 95% CI = 0.93–3.97), with significant heterogeneity (P = 0.000, *I*^2^ = 85.7%; Table 4, Fig 2). Four studies were conducted in Asia[6, 7, 10, 12]. Two studies performed adjustment for confounding factors[7, 10], and two studies did not adjust for confounding factors[6, 12]. After stratifying the analysis results by status of adjustment for covariates, we found no association between tooth loss and gastric cancer in the two studies adjusted for covariates (RR = 1.00, 95% CI = 0.68–1.47) and in the other studies unadjusted for covariates (RR = 3.54, 95% CI = 0.79–15.77). Only two studies controlled for the adjusted confounders[7, 10], such as smoking and alcohol drinking status; the combined RR was (RR = 3.54, 95% CI = 0.79–15.77). The sensitivity analysis showed unstable results, which ranged from 1.30 (95% CI = 0.82–2.07) when excluding the study of Demirer et al.[6] to 2.58 (95% CI = 1.03–6.46) when excluding the study of Hiraki et al.[7].

**Table 4:** Summary of results.

	Studies, N	RR (95%CI)	P-value	P of heterogeneity	*I*^2^ (%)
**Total**	8	1.44 (1.05–1.98)	0.025	0.001	71.3
**Study design**					
Case control study	4	1.92(0.93–3.297)	0.071	0.000	85.7
Cohort study	4	1.31(1.12–1.53)	0.001	0.615	0.0
**Case-control study**					
Geographic region					
Asia	4	1.92 (0.93–3.97)	0.077	0.000	85.7
Adjustment for covariates					
Yes	2	1.00 (0.68–1.47)	0.991	0.346	0.0
No	2	3.54(0.79–15.77)	0.098	0.001	90.9
**Cohort study**					
Geographic region					
United States	2	0.96 (0.56–1.64)	0.890	0.527	0.0
Asia	1	1.35 (1.14–1.59)	0.000	NA	NA
Europe	1	1.30 (0.75–2.62)	0.353	NA	NA
Adjustment for covariates					
Yes	3	1.33 (1.14–1.56)	0.000	0.843	0.0
No	1	0.77(0.32–1.85)	0.558	NA	NA
**Gastric cancer subsite**					
**Overall**	6	1.26(0.95–1.68)	0.104	0.373	6.8
Gastric cardia cancer	3	0.95(0.65–1.38)	0.785	0.801	0.0
Gastric non-cardia cancer	3	1.71(1.17–2.50)	0.005	0.912	0.0

RR, relative risk; CI, confidence interval; NA, not available.

The meta-analysis of all the four cohort studies indicated that tooth loss may be associated with increased incidence of gastric cancer (RR = 1.31; 95% CI = 1.12–1.53), with low heterogeneity (P = 0.615, *I*^2^ = 0.0%; Table 4, Fig 2). Two studies were conducted in the United States[4, 11], one in Europe [8], and one in Asia[5]. When stratified by geographic region, the association was stronger among the Asian population (RR = 1.35; 95% CI = 1.14–1.59) compared with that among the European (RR = 1.30; 95% CI = 0.75–2.26) and American populations (RR = 0.96; 95% CI = 0.56–1.64). Three studies adjusted for confounding factors[5, 8, 11], and only one study did not adjust for confounding factors[4]. The combined RR for gastric cancer was 1.33 (95% CI = 1.14–1.56) in studies controlled for confounding factors. Only two studies controlled the adjusted values, such as smoking and alcohol drinking status[5, 11]; the combined RR was 1.33 (95% CI = 1.13–1.57). Sensitivity analysis was also performed, in which one study was omitted at a time and the combined RR was calculated for the remaining studies. Results were similar to those in case-control studies. However, the association between tooth loss and gastric cancer was not statistically significant (RR = 1.11; 95% CI: 0.76–1.63) when excluding the study conducted by Abnet et al.[5]; in this study, a positive association was found between tooth loss and gastric cancer in the Chinese population.

### Tooth loss and incidence of gastric cancer subsites

**Fig 3:**
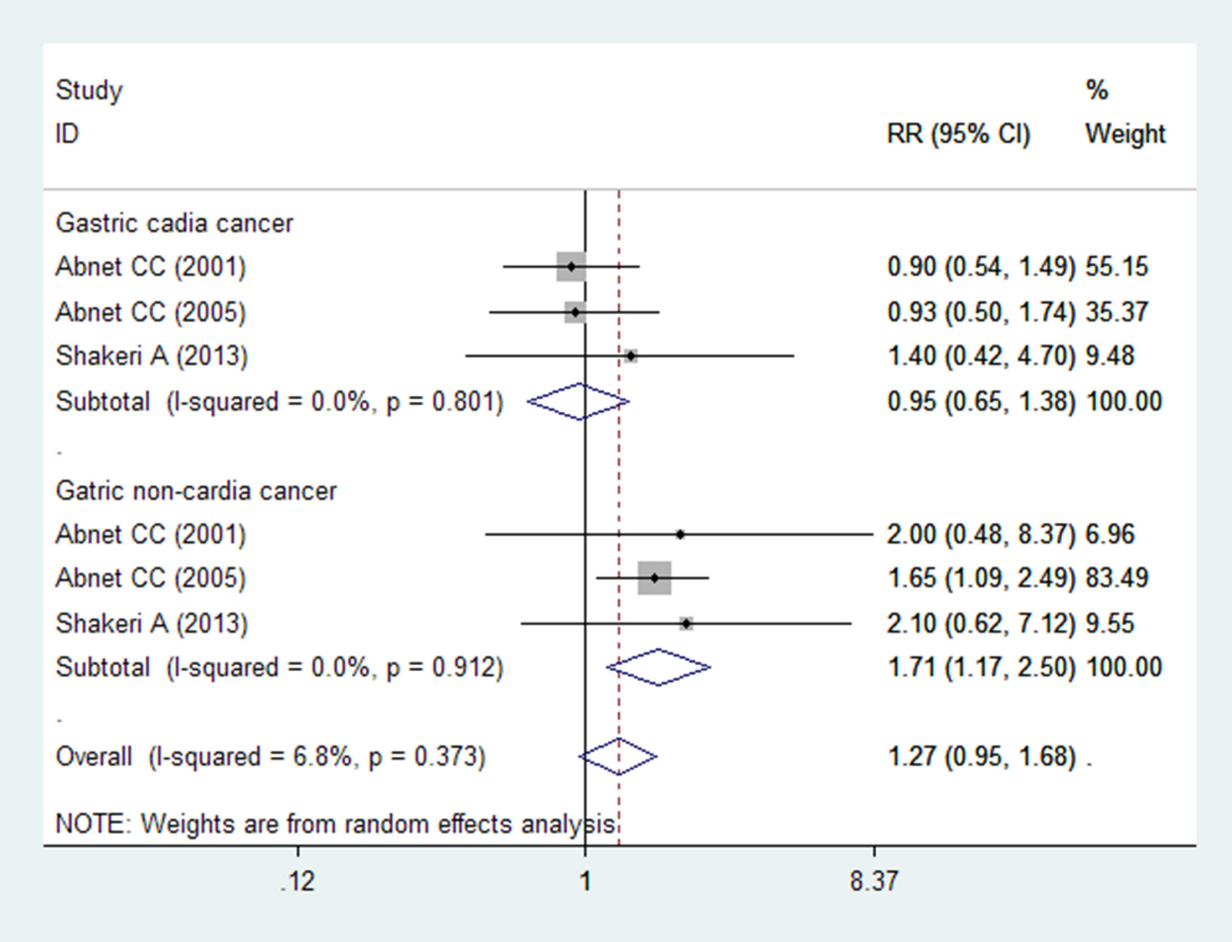
Forest plot for tooth loss and gastric cancer subtypes. Studies are pooled with the random-effects model.

Six studies (three articles) evaluated whether the association between tooth loss and incidence of gastric cancer differed by the anatomy of histological subsites; 1709 cases of gastric cardia cancers and 399 cases of gastric non-cardia cancers were obtained[8–10]. The pooled RR was 0.95 (95% CI = 0.65–1.38) for gastric cardia cancer and 1.71 (95% CI = 1.17–2.50) for gastric non-cardia cancer (Fig 3).

### Publication bias

**Fig 4:**
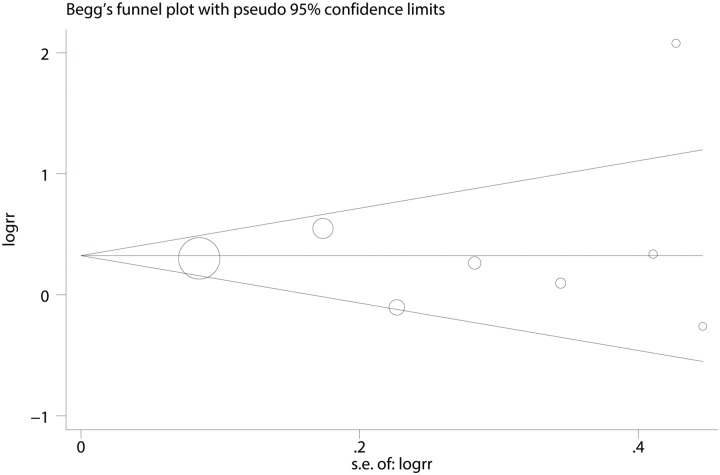
Begg’s funnel plot for publication bias analysis for tooth loss and gastric cancer.

The results of Begg’s and Egger’s funnel plot asymmetry tests (rank correlation test and regression method, respectively) in the meta-analysis indicated the absence of significant publication bias (Begg’s test, P = 0.90; Egger’s test, P = 0.74; Fig 4).

## Discussion

To the best of our knowledge, this meta-analysis is the first to explore the association between tooth loss and gastric cancer. The pooled results from the meta-analysis of eight observational studies by using the random-effects model suggested that tooth loss may be associated with increased incidence of gastric cancer.

The subgroup analysis showed that study design, geographic region, and adjustment for covariates may be the possible source of heterogeneity. Interestingly, the meta-analysis showed that the association was stronger for studies conducted in the Asian population than that for studies conducted in the American and European populations in included studies (Table 4). Therefore, populations with low socioeconomic status and different dietary patterns (mainly rice) may be at high risk for tooth loss and gastric cancer. Only one study provided OR estimates adjusted to socioeconomic status and showed that tooth loss is associated with increased incidence of gastric cancer[10]. In addition, infection with *H*. *pylori*, which can be found in periodontal pockets[24], may be a confounder for the possible relationship between tooth loss and gastric cancer risk. However, only one study from Finland[8] provided risk estimates adjusted for *H*. *pylori* infection and reported that tooth loss is associated with increased risk of gastric cancer.

The oral cavity, which provides a gateway between the external environment and the gastrointestinal tract, functions in food ingestion and digestion. Oral hygiene potentially affects the gastrointestinal flora and nutritional status and may thus have implications for the development of chronic diseases. Evidence shows that poor oral health or hygiene plays a role in cancer development, including cancer of the oral cavity and oropharynx[25], esophagus [23, 26], lung [11], pancreas[27], and kidney[11]. Dental caries is the primary cause of tooth loss in adults[28, 29]. The primary cause of caries in the general population is carbohydrate intake, which is a potential cofounder[30, 31]. Carbohydrate intake can also damage health through various mechanisms[32]. Carbohydrate-rich foods are the main dietary component that affects insulin secretion and glycemic responses in an individual[33]. Furthermore, evidence indicates that insulin resistance and chronic hyperinsulinemia are implicated in the etiology of gastric cancer[34–36]. In particular, chronic hyperinsulinemia can increase the bioactivity of insulin-like growth factor 1[37], which stimulates tumor development by inhibiting apoptosis and stimulating tumor cell proliferation[38].

The present meta-analysis exhibits three strengths. First, this meta-analysis is the first to investigate the association between tooth loss and gastric cancer. Second, the relatively large number of participants improved the precision of risk estimates. As such, conclusions based on this meta-analysis were well founded. Third, most of the included studies used multivariable-adjusted risk estimates, thereby minimizing confounding factors.

This meta-analysis presents several limitations that must be considered in interpreting the results. First, observational studies have intrinsic limitations, such as selective bias and recall or memory bias. Moreover, case-control studies cannot establish exposure prior to gastric cancer diagnosis. This limitation can partly explained the different results between case-control and cohort studies in the stratified analysis. Second, confounders controlled in the included studies differed, which may also explain the inconsistent results. Most studies adjusted for major risk factors (e.g., age, sex, education, smoking status, and alcohol consumption), and few studies controlled for infection with *H*. *pylori* and socioeconomic status. None of the studies controlled for carbohydrate intake. The inconsistency in adjustment for confounders across all studies is a limitation of this meta-analysis that cannot be quantified. Third, significant heterogeneity was detected. Heterogeneity among studies should not be ignored even if it is highly common in meta-analysis. Studies included in this meta-analysis are heterogeneous in terms of different populations investigated and diagnostic criteria for tooth loss, thereby contributing to the heterogeneity in the pooled analysis. Furthermore, unstable results were observed in subgroup and sensitivity analysis, which indicated that more relevant articles are needed to further explore this association. Fourth, the findings are likely to be influenced by misclassification of exposure because the majority of studies employed different methods to assess and categorize tooth loss. Therefore, the results should be considered with caution because of exposure misclassification. Overall, these limitations may affect our final conclusions.

This meta-analysis suggests that patients with tooth loss have increased incidence of gastric cancer. However, we can not concluded in the meta-analysis that tooth loss may be a risk factor for gastric cancer due to significant heterogeneity among studies and mixed results between case-control studies and cohort studies. Additional large-scale and high-quality prospective studies are needed to evaluate the association between tooth loss and risk of gastric cancer.

## Supporting Information

S1 PRISMA Checklist (DOC)
